# ATP‐Powered Signaling Between Artificial and Living Cells

**DOI:** 10.1002/anie.202517843

**Published:** 2025-10-18

**Authors:** Soumya Sethi, Charu Sharma, Andreas Walther

**Affiliations:** ^1^ Life‐like Materials and Systems Department of Chemistry University of Mainz Duesbergweg 10–14 55128 Mainz Germany; ^2^ Department of Bionanoscience Kavli Institute of Nanoscience Delft University of Technology Delft The Netherlands

**Keywords:** Artificial cell signaling, Artificial cell–living cell communication, ATP‐fueled reaction network, ATP‐responsive biomaterials, Dissipative reaction network

## Abstract

ATP is the energy currency of life and is overabundant in the tumor microenvironment, where it has been suggested as a target for cancer therapy. We introduce ATP‐dissipative delivery of DNA signals from artificial cells to living cells by exploiting an ATP‐driven reaction network that transiently ejects DNA Signal strands from the shielded artificial cell interior to the extracellular medium of living cells. We customize the Signal for intracellular uptake or for extracellular instruction using a cytokine‐ssDNA chimera that can trigger efficient intracellular downstream signaling programs. Our study discusses details of system design on a timer circuit and artificial cell level, system integration challenges, and how ATP concentrations regulate the transient delivery. The strategy can be extended to deliver therapeutic oligonucleotides for applications in gene therapy and gene silencing. For cancer therapy, it can use naturally enhanced ATP levels to induce selective delivery of therapeutic oligonucleotides.

## Graphical abstract

We introduce an ATP‐powered artificial cell platform capable of selectively and transiently releasing DNA signals to facilitate communication between artificial and living cells. By engineering these DNA signals, we achieve precise activation of cellular functions and regulated delivery in response to ATP levels.

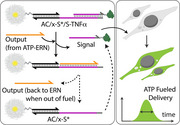

Interfacing artificial cells (AC) and mammalian cells can facilitate the development of programmable therapeutics that can sense, process, and respond to physiological changes,^[^ ^1^, ^2^ ^]^ create biohybrid systems^[^ ^3^, ^4^ ^]^ for regenerative medicine,^[^ ^5^ ^]^ enable targeted drug delivery,^[^ ^6^, ^7^, ^8^ ^]^ or allow artificial tissue engineering.^[^ ^9^, ^10^, ^11^ ^]^ To establish effective communication between artificial and living cells, direct cellular contact as well as (bio)chemical signaling systems have been considered.^[^ ^12^ ^]^ Contact‐dependent communication relies on direct physical interactions at its interfaces, enabling specific signal exchange.^[^ ^13^ ^]^ These interactions can be engineered using programmable systems, relying on DNA self‐assembly, which allows ACs to physically contact and recognize living cells.^[^ ^14^ ^]^ Besides this, the use of engineered signaling mechanisms^[^ ^15^ ^]^ has been demonstrated, for instance, ACs encapsulating transcription‐translation machinery to instruct mammalian cells.^[^ ^16^, ^17^ ^]^

ATP is a molecule of interest for communication among artificial and mammalian cells due to its dual roles as both energy currency^[^ ^18^ ^]^ and signaling molecule.^[^ ^19^ ^]^ ATP is recognized as one of the main biochemical components of the tumor microenvironment (TME) with concentrations in the range of 50–200 µM.^[^ ^20^, ^21^, ^22^ ^]^ Depending on its concentration in the TME, the presence of ATP‐hydrolyzing enzymes and receptors expressed by cancer and immune cells, tumor cell proliferation can be promoted or suppressed.^[^ ^23^ ^]^ With increased understanding of information exchange in TME, new therapeutic approaches can be developed to directly target extracellular ATP and the TME. In the context of ACs, this requires a biocompatible AC system that can release bioactive components of choice in response to ATP.

Building on ATP‐fueled enzymatic reaction networks (ERNs),^[^ ^24^, ^25^, ^26^ ^]^ here, we design an ATP‐fueled DNA delivery system that transiently releases Signals (single‐stranded ssDNA strands) into the extracellular media of cancer cells (Figure 1). The system is composed of an ATP‐driven ERN and ACs bearing the Signal to be delivered to target cells. The AC has the key function to shield the Signal against non‐specific internalization by cells by harboring it in its interior. In the presence of ATP, the fuel‐driven ERN is activated to temporarily provide the Signal to the cells. Once ATP is consumed, the ATP‐ERN resets the entire system to its initial state, which involves the recapturing of excess Signal by ACs. This enables controlled, ATP‐dependent Signal delivery. Since HeLa cells alone do not produce sufficient ATP,^[^ ^27^, ^28^ ^]^ we exogenously add ATP to drive the system at concentrations found in the TME.^[^ ^20^, ^21^, ^22^ ^]^ We further extend this approach by coupling the Signal strand with a protein‐DNA chimera for targeted delivery of cell‐signaling cytokines. Importantly, this ATP‐driven system actively consumes ATP, enabling self‐regulated delivery responsive to ATP levels comparable to those present in the TME.

**Figure 1:**
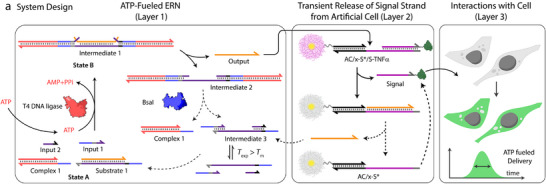
System design for controlled delivery of DNA in living cells via out‐of‐equilibrium extracellular medium.

In more detail, our system has three different layers: Layer 1 contains the ATP‐fueled ERN, which operates as a molecular control program for the transient release of Signal from ACs in Layer 2, which is followed by uptake of Signal by living cells in Layer 3. The ATP‐driven ERN builds on our initial design^[^ ^16^, ^17^, ^18^, ^19^, ^20^, ^21^ ^]^ of a DNA‐based ligation/restriction ERN that, in the present configuration, enables the ATP‐powered transient release of a ssDNA that is recaptured once ATP is consumed in the ligation/restriction process. The ERN‐to‐AC integration sets our design apart from recent work on ATP‐instructed cell communication.^[^ ^29^ ^]^ In its initial deactivated State A, the ERN is composed of two double‐stranded (ds) DNA complexes (Complex 1, Substrate 1) and two ssDNA (Input 1, Input 2; Figure 1). In this state, Inputs 1 and 2 are incapable of displacing Output from Substrate 1 due to unstable hybridization of Input 1 (melting temperature (*T*_m_) = 32 °C < experimental temperature (*T*_exp_) = 37 °C, by NUPACK^[^ ^30^ ^]^) and Input 2 (*T*_m_ = 22 °C < *T*_exp_ = 37 °C) with a longer strand of Substrate 1. However, the addition of ATP powers the covalent ligation of Substrate 1 with two molecules of Complex 1 and one molecule each of Inputs 1 and 2 with the help of T4 DNA ligase, generating Intermediate 1. The system is pushed towards an activated transient state, where strand displacement from two sides now provides a strong thermodynamic push to release Output from Intermediate 1, finally generating Intermediate 2. The dual invasion strategy is essential for releasing an Output strand long enough to displace Signal in Layer 2.^[^ ^31^, ^32^, ^33^ ^]^ Concurrently, BsaI cleaves Intermediate 2 to regenerate Complex 1 and Intermediate 3. Because of low *T*_m_ (32 °C < *T*_exp_ = 37 °C), Intermediate 3 dissociates into Input 1 and Input 2, and reproduces Substrate 1 after re‐hybridization with Output, returning the system to State A. Faster ligation than cleavage satisfies the kinetic boundary conditions required for the system to achieve a dynamic steady state (DySS) with an ATP‐dependent lifetime of the Output before the restriction overtakes and brings the system back to its initial state. ^[^ ^16^, ^17^, ^18^, ^19^, ^20^, ^21^ ^]^ The Output is therefore generated in an uphill fashion and is transiently available in the system to perform downstream functions before it is ultimately reassociated with Substrate 1.

We use the transiently released Output as a timer circuit to displace the Signal from an AC, hence conferring dissipative properties of the Output to the cell‐instructive Signal. Once restriction overtakes ligation, the Output prefers to regenerate Substrate 1 rather than residing with the AC (see NUPACK calculations in Figure S1). As a result, the AC can also recapture the Signal, which reforms the initial state of the system. The lifetime and amount of the Signal released in the DySS depend not only on the ATP concentration but are also limited by the transient Output yield. The Signal can be designed to be internalized by mammalian cells, and is therefore lost for recovery, or it can be coupled to a cytokine to instruct cells extracellularly.

Interfacing this ATP‐driven ERN with cells requires addressing several integration challenges. First, we require a cell‐signaling strand that can conditionally be made available for cells and that remains “hidden” when not in the activated state to prevent unwanted signaling. We chose to immobilize it on ACs based on core‐shell microgels^[^ ^34^ ^]^ that provide easy diffusive access for DNA strands for strand displacement, while at the same time shielding it in the absence of the Output messenger strand. Additionally, the colloidal nature simplifies analytics in a CLSM during release and recapture. Second, for intracellular delivery, we require a Signal able to cross cell membranes in detectable amounts. Thus, we screened ssDNAs with different dyes, which are known to facilitate entry due to their hydrophobicity, and identified that Atto 645N and Cy5‐labeled ssDNA are readily taken up by HeLa cells and localize to the mitochondria, whereas Atto 488‐ssDNA is not taken up (Figure S2).^[^ ^35^, ^36^, ^37^, ^38^ ^]^ Third, it is pivotal to suppress DNAse‐mediated degradation of the DNA components by high nuclease concentrations present in typical cell culture medium containing fetal bovine serum (FBS).^[^ ^39^ ^]^ We optimized a dedicated cell culture medium to provide stability for over one day, whereas FBS‐containing media show significant degradation within 4 h (Figure S3).

Before instructing HeLa cells, we turn to the integration of the ATP‐ERN with ACs. For this, we functionalized micron‐sized core‐shell microgels^[^ ^34^, ^40^ ^]^ as membrane‐less AC chassis in their crosslinked PNIPAM‐co‐AA hydrogel shell with NH_2_‐ssDNA through EDC‐mediated coupling (84.2 wt% N‐isopropylacrylamide (NIPAM); 10.4 wt% acrylic acid (AA); 1 wt% N,N’‐methylenebis(acrylamide) (MBA), EDC = 1‐Ethyl‐3‐(3‐dimethylaminopropyl)carbodiimide; Figure 2). This leads to a typical DNA grafting density of 3.3 × 10^6^ strands per AC or 132 ± 5 µmol strands per g of AC (Figure S4). Due to the strong negative zeta potential of the ACs (ζ = −18 ± 2 mV) and the large size, such ACs cannot be internalized by cells within 24 h of incubation (Figure S4). Atto 647N‐labeled Signal strand (S‐Atto 647N) is immobilized with the help of a Linking strand (*x*‐S*Q) to a final concentration of 3.75 µM (S‐Atto 647N) on ACs with pendant x* ssDNA. CLSM confirms successful functionalization by the appearance of bright fluorescence in the Atto 647N channel (Figure 2b). This strategy offers easy tuning of the amount of Signal on ACs.

**Figure 2:**
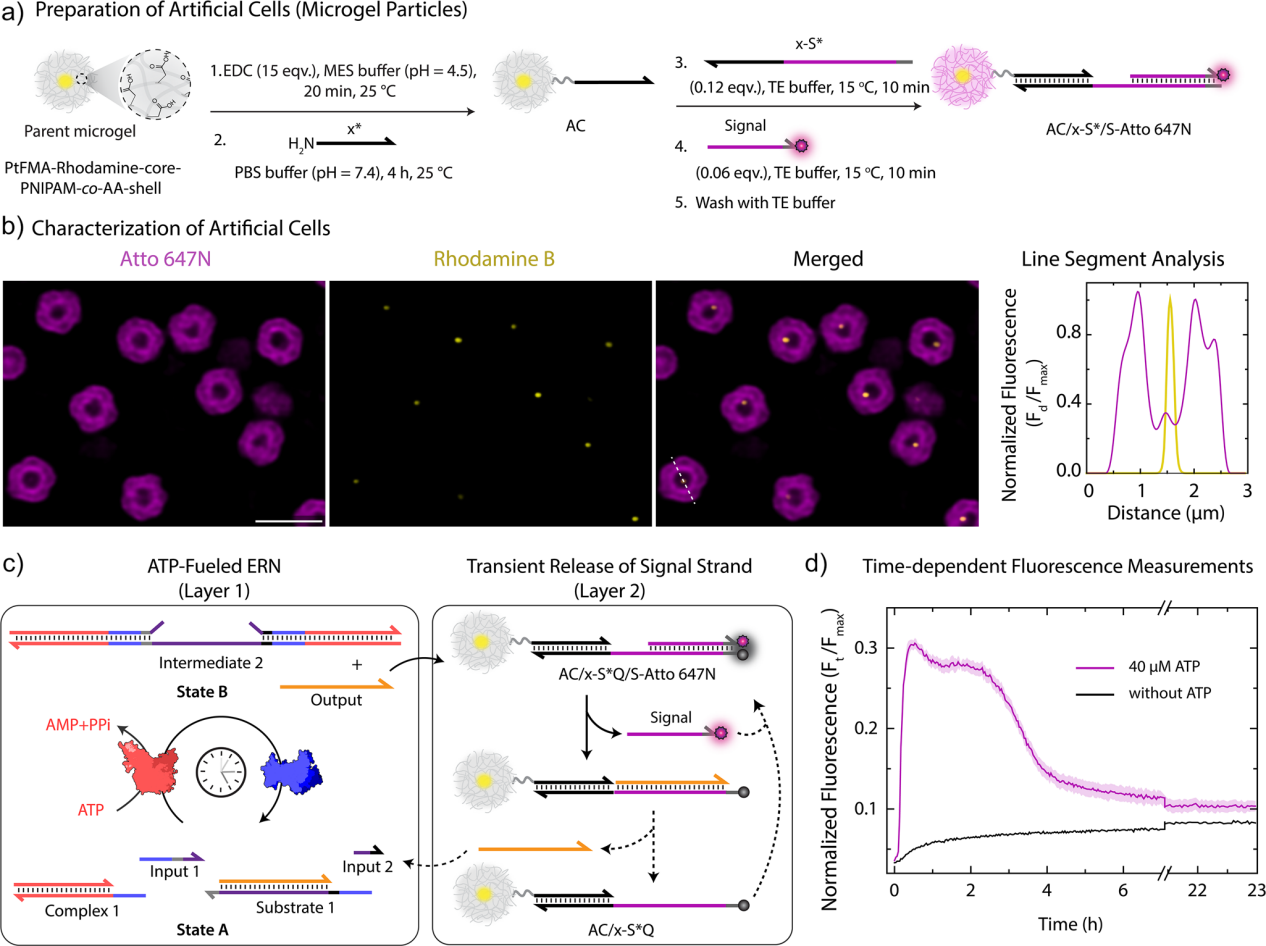
Preparation of DNA‐functionalized ACs for the transient release of Signal. a) Scheme depicting stepwise preparation of AC/x‐S*/S‐Atto 647N from parent AC. b) CLSM characterization: AC/x‐S*/S‐Atto 647N suspended in TE buffer (pH = 8.0) at 0.05 wt%. c) Schematic representation of transient release of Signal from AC/x‐S*Q/S‐Atto 647 N via ATP‐fueled ERN. d) Time‐dependent FI changes demonstrate the transient increase of fluorescence because of Signal strand release into the medium upon ATP addition. Results are normalized with respect to 3.75 µM of S‐Atto 647 N (Signal) strands dissolved in DNA–Cell buffer, which corresponds to the maximum fluorescence that can be observed in the system, average of two measurements. The shaded region depicts the standard deviation (SD) of triplicates. Scale bar: 3 µm.

Next, we coupled Signal‐containing ACs to an ATP‐fueled ERN to control the transient release of Signal in a dissipative fashion (Figure 2c). Experimentally, ATP‐fueled ERNs are optimized to operate at maximum efficiency in 1X Cut Smart buffer in Milli Q water.^[^ ^24^, ^33^ ^]^ However, to ensure the eventual coupling with cells, we simply prepared the 1X Cut Smart buffer in Cell buffer. We then checked the performance of this DNA–Cell buffer for transient Signal generation. To simplify monitoring via fluorescence intensity (FI) changes, we further exchanged the linking strand *x*‐S* to a quencher‐modified *x*‐S*Q to monitor release of S‐Atto 647N by appearance of fluorescence. Next, we assembled Signal‐containing ACs (3.75 µM; 0.05 wt%) and the ATP‐driven ERN in DNA‐Cell buffer at 37 °C with 20 µM Complex 1, 5 µM Substrate 1, 10 µM Input 1, and Input 2 using 0.8 Weiss units (WU) µL^−1^ of T4 DNA ligase and 0.8 units (U) µL^−1^ of BsaI, and initiated the ERN with 40 µM ATP. Before ATP addition, initial fluorescence is low, confirming the efficient quenching of the S‐Atto 647N by the quencher (Figure 2d). Once ATP is present, an immediate increase in FI by ~ 30% occurs, signifying release of the Signal. This is followed by a DySS of ca. 2.5 h, where ATP‐fueled ligation and restriction occur in balance. Once the system runs out of ATP, BsaI‐controlled restriction takes over, and the Output, temporarily scavenged in AC on AC/x‐S*Q/Output, returns to Layer 1 and reforms Initial State A. This makes the AC‐x‐S* available for Signal again, causing its recapture and drop of the FI. In previous works, we have shown how ATP can tune the lifetime of such systems and allow for reinitiation.^[^ ^25^, ^31^, ^32^ ^]^ Figure S5 shows exemplary tuning of lifetime by injecting 80 µM ATP, leading roughly to a doubling of the lifetime—as expected, along with re‐activation at 40 µM ATP.

Understanding the amount of Signal transiently ejected from ACs is an important aspect. NUPACK simulations suggest that mixing 5 µM Output with 3.75 µM Signal complex present on the ACs (x*/*x*‐S*/S = 7.5/7.5/3.75 µM) releases 3.5 µM Signal (Figures S1 and S6), corresponding to 94% yield. Experimentally, direct addition of Output produces 3.52 µM Signal. In comparison, ATP‐fueled Output generation, using 40 µM ATP, yields ca. 1.47 µM (39%) of Signal (details in Figure S7) due to the DySS nature of the system. From previous experiments for cellular uptake of dye‐labeled ssDNA (Figure S2), we know that this concentration is sufficient to efficiently transfer Signal into HeLa cells within 2.5 h. Note that a minimal increase of ca. 6% in the absence of ATP is caused by unavoidable leakage between Substrate 1 and the Signal complex in the ACs (Figure S7). However, this increase in fluorescence is slow and insufficient to cause any observable cellular internalization.

Building on the understanding of the transient ATP‐driven release from ACs, we next combined it with cellular delivery using HeLa cells as a model cell line (Figure 3). CLSM helps to understand the details of the transfer process (Figure 3b). We prepared Signal‐containing ACs, but omitted the quencher on the Linker strand, enabling tracking of Signal transfer from ACs to the cells all the way through the medium upon ATP addition. With ACs in hand, we integrated the same ATP‐fueled ERN as above and triggered it with 40 µM ATP. Before adding ATP, ACs show high fluorescence due to the bonded Signal, and no fluorescence can be detected within cells marked with white dotted lines (Figure 3b,c). However, within 18 min of ATP addition, FI on ACs decreases by 90%, and a concurrent FI increase can be detected in the medium, attaining a transient maximum FI (Figure 3c). Notably, during the next 2.5 h, the FI in the medium decreases, whereas the FI of the cells increases, and the FI of the ACs remains stable. This clearly demonstrates that in this DySS, Signal is being transferred from the ATP‐fueled process into cells. Once ATP is consumed (ca. at 2.5 h, Figure 3d), ACs begin to recover their FI by recapturing excess Signal from the medium. A partial recovery of only 37% in FI on the ACs confirms partial internalization of Signal by cells, which reach a similar FI at the end. Note that S‐Atto 647N localizes to the mitochondria of cells. Upon comparing the FI decrease on ACs with the FI increase of the medium at 24 h of ATP addition, we estimate the amount of Signal internalized within cells during this time to be 2.06 µM or 1.24 x 10^11^ Signal strands per cell (see Figure S7 for further details). A control system without ATP shows only minor Signal transfer due to minor strand displacement leakage (Figure 3c (left), Figure S8). Cell viability remains high with an overall decrease of only 12% after 24 h (Figure S11).

**Figure 3:**
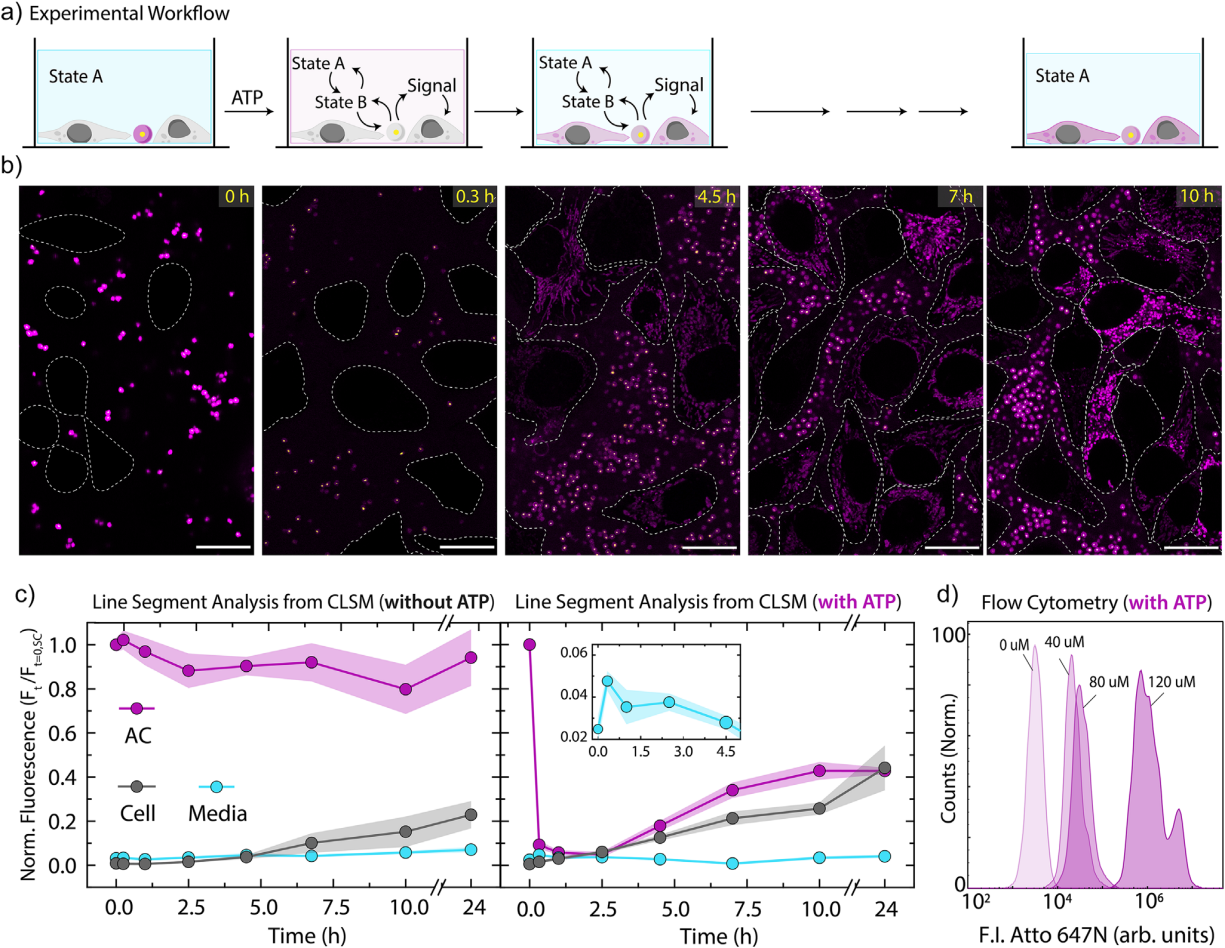
ATP‐fueled Signal transfer from ACs to HeLa cells. a) Scheme of experimental workflow where Layer1 and Layer2 are installed within the extracellular medium of cells marked by initial State A. State B is acquired upon ATP addition, which marks the transient release of Signal strands from ACs into the medium to be uptaken by cells. b) CLSM imaging of ATP‐fueled delivery of Signal from ACs into cells. Dotted lines define the cell boundary. c) Time‐dependent FI changes on ACs, in medium and within cells without and with ATP. Inset shows the zoomed‐in region of the FI changes in the media. The fluorescence values represent the maximum intensity acquired from line segment analysis and normalized with respect to the maximum fluorescence observed on ACs. Results represent the average contribution from five different regions from three replicates, and shaded regions depict SD. d) Flow cytometer measurement of cells in the presence of ATP‐fueled ERN networks at various ATP concentrations to study the release and uptake of dye‐labeled Signal. Scale bars: 20 µm.

More importantly, we quantitatively assessed the percentage of cells that incorporate the Signal under varying ATP concentrations, from 0 to 120 µM, using flow cytometry at the 30‐min time point. In the absence of ATP, cells exhibit minimal uptake of Signal, resulting in the lowest Atto 647N FI. As expected, at ATP concentrations of 40, 80, and 120 µM, the percentage of positive cells is high with a slight increase from 97.6% to 99.9% (Figure S10). However, the extent of delivered Signal is strongly different as the FI per cell correspondingly increases with higher ATP concentrations. (Figure 4d). Furthermore, we analyzed cells at fixed ATP concentrations (40 and 80 µM) across different time points (0.5, 2, and 4 h). As time progresses, cells take up more Signal, and the FI within cells increases accordingly (Figures S11 and S12).

**Figure 4:**
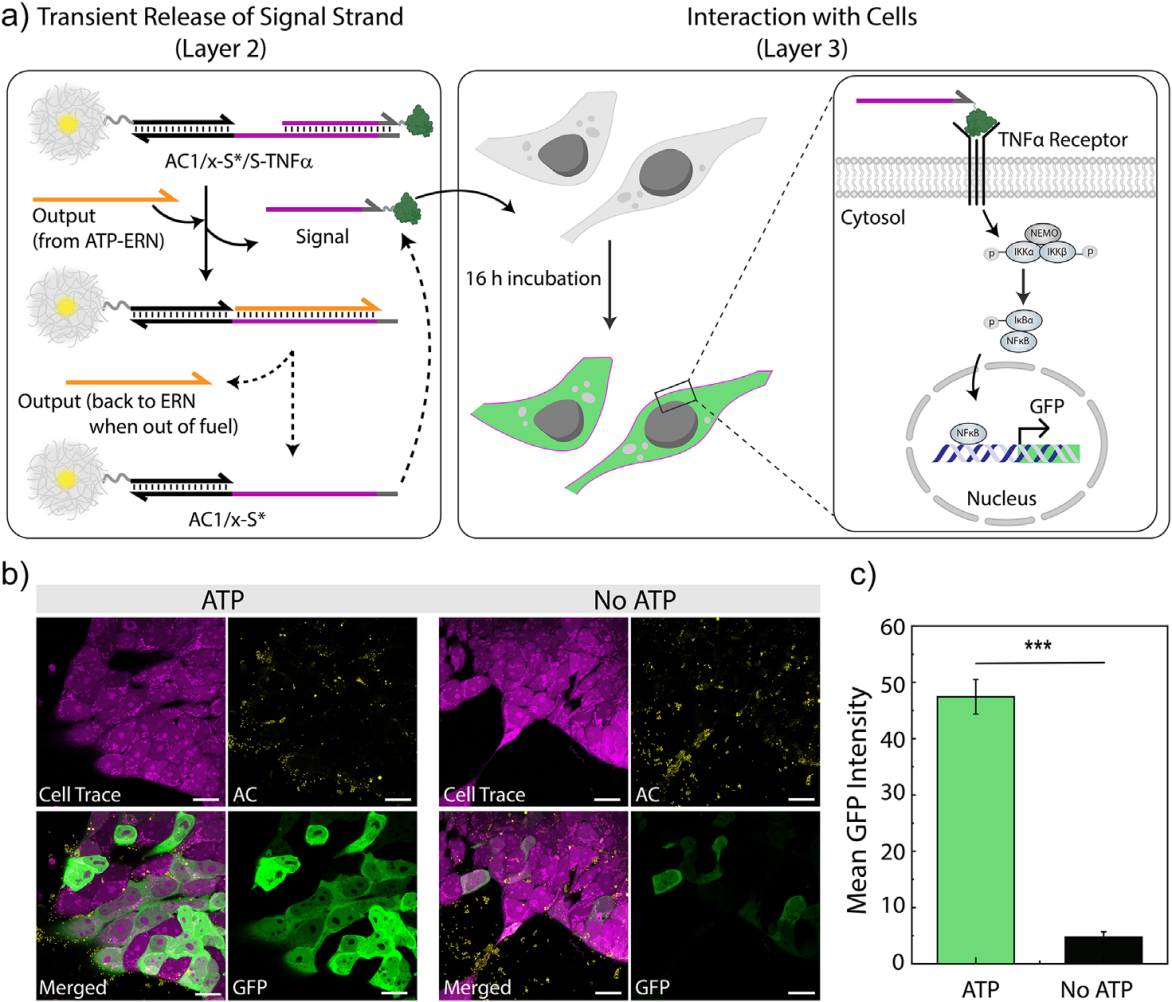
Cellular response to ATP‐fueled release of TNFα‐linked ssDNA Signal. a) Simplified scheme illustrating the incubation of cells and ACs in ATP‐fueled environments. b) CLSM imaging of GFP fluorescence expressed by the TNFα‐RCL in the absence and presence (40 µM) of ATP after 16 h. c) Quantified mean GFP intensity from CLSM images of cells in the presence and absence of ATP. The results are presented as the mean ± SEM, *n* = 72 cells from three replicates. Statistics: Unpaired, two‐tailed student's *t*‐test. Scale bars: 20 µm.

Directing cellular behavior with the ATP‐fueled ERN system is critical for applications in cancer biology,^[^ ^41^ ^]^ where modulation of extracellular signals provides an entry point for therapy. Thus, we investigated whether our system can be used to trigger programmed release of a cytokine to affect cellular function. To do so, we modified our approach for the Signal strand and conjugated TNFα (tumor necrosis factor) protein to it (Figure S13 shows a similar transient lifetime for the ejection of the TNFα Signal strand as compared to the Atto 647N Signal strand used above). TNFα is an important TME signal to promote cancer cell death and neoantigen presentation, overall promoting immune response in cancer. To understand whether ATP‐dissipating signaling can be exploited for extracellular signaling, we employed a reporter cell line, i.e., NF‐кB/293/GFP‐Luc Transcriptional Reporter Cell Line (TNFα‐RCL), to analyze the nuclear factor Kappa B (NF‐кB) pathway. In the presence of TNFα ligand, TNFα‐RCL generates GFP in a concentration‐dependent fashion, readily detected by CLSM (Figure 4a).^[^ ^42^ ^]^ We first examined the effect of native TNFα on TNFα‐RCL and observed that TNFα‐RCL produced GFP fluorescence (Figure S14). Then, we assessed the efficacy of TNFα‐ssDNA conjugate by incubating with TNFα‐RCL in DNA–Cell Buffer. Indeed, TNFα‐RCL produces GFP fluorescence (Figure S15), confirming that TNFα‐RCL thrives in DNA–Cell Buffer and TNFα‐ssDNA conjugate signals similarly to a native TNFα. Next, we investigated the time point of GFP production in TNFα‐RCL, noting that 16 h after dosing is sufficient for GFP production (Figure S16). Finally, we assembled our AC systems with S‐TNFα together with the upstream ATP‐fueled ERN, along with TNFα‐RCL (Figure 4a). Adding ATP as a fuel leads to a transient S‐TNFα release, indeed triggering the NF‐кB pathway. This results in a ten‐fold higher GFP fluorescence with respect to the control (no ATP), where GFP expression is close to zero (Figure 4b,c). These findings demonstrate that our ATP‐driven reaction network is both robust and adaptable, capable of functioning in the extracellular environment of cells and can be engineered to release protein‐linked DNA strands on demand to trigger cellular pathways.

In summary, we have demonstrated an ATP‐powered AC system to control the transient delivery of DNA signals from a shielded AC configuration to living cells. ATP consumption drives Signal release and its eventual recapture, imparting temporal control and dissipative behavior to the signaling process. Dissipative behavior returns the system back to its original state once the fuel is exhausted, giving rise to homeostatic control mechanisms. For the successful operation of longer‐lasting ERNs, we customized the media to maintain the stability of all DNA‐based components. Since the rate of DNA uptake by cells is a cell‐intrinsic parameter, the rate of DNA release and recapture from the upstream ERN needs to be programmed to match uptake kinetics. This signal can either be engineered for uptake by mammalian cells–rendering it unrecoverable–or linked to a cytokine to act externally and guide cellular behavior, where the reporter cell line provided clear evidence for successful cytokine instruction. Due to the modularity of our design, therapeutic oligos such as siRNA, miRNA, and antisense oligos may be delivered into cells to enable advanced applications in gene therapy and gene silencing. Fuel concentrations of 50–200 µM have been detected in TME, which is commensurate with the ATP‐ERN system presented here. A future realistic AC‐based delivery platform needs to immobilize enzymes and all other components of the ERN inside a fully self‐contained AC to allow, for instance, peritumoral application.

## Supporting Information

The authors have cited additional references within the Supporting Information.^[^ ^40^, ^42^, ^43^, ^44^ ^]^

## Conflict of Interests

The authors declare no conflict of interest.

## Supporting information



Supporting Information

## Data Availability

The data that support the findings of this study are available from the corresponding author upon reasonable request.
